# Assembly of the Respiratory Mucin MUC5B: A NEW MODEL FOR A GEL-FORMING MUCIN*

**DOI:** 10.1074/jbc.M114.566679

**Published:** 2014-04-28

**Authors:** Caroline Ridley, Nikos Kouvatsos, Bertrand D. Raynal, Marj Howard, Richard F. Collins, Jean-Luc Desseyn, Thomas A. Jowitt, Clair Baldock, C. William Davis, Timothy E. Hardingham, David J. Thornton

**Affiliations:** From the ‡ Wellcome Trust Centre for Cell-Matrix Research and; the § Faculty of Life Sciences, The University of Manchester, Oxford Road, Manchester M13 9PT, United Kingdom,; the ¶ INSERM U995, University of Lille, F-59045 Lille, France, and; the ‖ Cystic Fibrosis/Pulmonary Research and Treatment Center, University of North Carolina, Chapel Hill, North Carolina 27517-7248

**Keywords:** Analytical Ultracentrifugation, Cystic Fibrosis, Mucin, Mucus, Recombinant Protein Expression, Single Particle Analysis, Goblet Cell

## Abstract

Mucins are essential components in mucus gels that form protective barriers at all epithelial surfaces, but much remains unknown about their assembly, intragranular organization, and post-secretion unfurling to form mucus. MUC5B is a major polymeric mucin expressed by respiratory epithelia, and we investigated the molecular mechanisms involved during its assembly. Studies of intact polymeric MUC5B revealed a single high affinity calcium-binding site, distinct from multiple low affinity sites on each MUC5B monomer. Self-diffusion studies with intact MUC5B showed that calcium binding at the protein site catalyzed reversible cross-links between MUC5B chains to form networks. The site of cross-linking was identified in the MUC5B D3-domain as it was specifically blocked by D3 peptide antibodies. Biophysical analysis and single particle EM of recombinant MUC5B N terminus (D1D2D′D3; NT5B) and subdomains (D1, D1-D2, D2-D′-D3, and D3) generated structural models of monomers and disulfide-linked dimers and suggested that MUC5B multimerizes by disulfide linkage between D3-domains to form linear polymer chains. Moreover, these analyses revealed reversible homotypic interactions of NT5B at low pH and in high calcium, between disulfide-linked NT5B dimers, but not monomers. These results enable a model of MUC5B to be derived, which predicts mechanisms of mucin intracellular assembly and storage, which may be common to the other major gel-forming polymeric mucins.

## Teaser

**Background:** Mucin polymer formation is a complex intracellular process.

**Results:** MUC5B N-terminal D3-domains form reversible pH-sensitive calcium mediated cross-links between linear MUC5B polymer chains.

**Conclusion:** Intracellular assembly of MUC5B generates disulfide-bonded polymers which form calcium mediated condensed networks in secretory granules.

**Significance:** This identifies a new model for mucin assembly that may be common to other polymeric mucins.

## Introduction

Mucus is a viscoelastic gel that is secreted by epithelial cells to protect the epithelium from environmental factors (1). The biological and physical properties of this mucus layer are essential for its protective function, and disruption of mucus organization results in pathology. In the gastrointestinal tract, depletion of the mucus barrier results in increased tumor formation and chronic infection with intestinal dwelling nematodes (2, 3). In contrast, accumulation in the airways of mucus with abnormal viscosity is a central pathological feature of cystic fibrosis, asthma, and chronic obstructive pulmonary disease (4, 5). Details of the molecular basis of mucus gel properties and function need to be better understood to elucidate the mechanisms underlying these pathological changes.

MUC5B is a major gel-forming mucin in respiratory tract mucus and is indispensable for mucociliary clearance that controls bacterial infection (6). It is a member of a family of high molecular mass (2–50 MDa) polymeric glycoproteins (other members are MUC2, MUC5AC, MUC6, and possibly MUC19) that form the matrix of epithelial mucus gels. Polymeric mucins are encoded by closely related genes, which are selectively expressed in specialized epithelial cells. They are assembled intracellularly and stored in granules prior to secretion, but the molecular mechanisms involved in their assembly into polymers are not fully understood (7). Polymeric mucins are glycoproteins in which up to 80% of the total mass is carbohydrate. The protein structure of this gene family of mucins shares major features, including central extended regions consisting of serine- and threonine-rich repeats (glycosylated or mucin domains), which form the site of dense *O-*linked glycan substitution, and these glycosylated regions are interrupted centrally and capped at the N and C termini by folded cysteine-rich domains (1). The N-terminal (D1-D2-D′-D3) and C-terminal (D4-B-C-CK) cysteine-rich domains are both involved in mucin polymerization through disulfide bridges. Mucins share some sequence similarity in these N- and C-terminal domains to the glycoprotein, von Willebrand factor (vWF)^2^ (8, 9), which also assembles into polymeric forms. The assembly of vWF through N- and C-terminal disulfide bridges into a core head-to-head and tail-to-tail linked polymer structure is well documented (10–12), and studies on intact mucins and recombinant N- and C-terminal domains of MUC2, MUC5AC, and porcine submaxillary mucin have shown that these gel-forming mucins share some of this basic pattern of disulfide-linked assembly with vWF (13–16). However, the later stages of assembly involving multimerization and packaging into secretory granules, which help define the secreted product and its epithelial function, are less clearly resolved.

Prior to secretion, the assembled mucin polymers are packaged in a highly condensed and dehydrated state inside secretory granules within the epithelial goblet cells or glandular mucous cells in which they are produced. It is at this stage that Ca^2+^ ions are proposed to charge-shield the sialic acid and sulfate groups on the mucin glycans to help attain a condensed and dehydrated state (17, 18). Additionally, the calcium-dependent cross-links that we have previously identified between MUC5B mucins through protein sites on the polypeptide (19) might also be active in this packaging process.

Recent evidence has shown that mucins are highly organized within the secretory granule and that this is dramatically changed as the mucin is released onto the epithelial surface (20, 21). The mechanism that controls this transition during secretion from the condensed packaged mucin to the expanded gel form remains to be fully elucidated, but it is clear that during secretion there is a large change in the ionic conditions, with a sharp rise in pH and a decrease in Ca^2+^ concentration, and these have been proposed to help drive the unfolding mechanisms (20). To define the molecular detail of MUC5B intracellular assembly and packaging in secretory granules, we investigated (*a*) the interactions of Ca^2+^ with intact polymeric MUC5B; (*b*) the mechanism of MUC5B polymeric assembly using MUC5B expressed recombinant N-terminal protein domains; and (*c*) the effect of Ca^2+^ and pH on the multimerization of N-terminal protein domains.

## EXPERIMENTAL PROCEDURES

### 

#### 

##### Preparation of “Native” MUC5B

Fresh whole human saliva (six different donors) was solubilized in 0.1 m NaCl, 20 mm Tris, pH 7.4, and native MUC5B mucins were purified by CsCl density gradient centrifugation and Sepharose CL-2B size exclusion chromatography as described previously (19). Samples of purified native MUC5B mucins were labeled with fluorescein isothiocyanate (FITC) as described previously (22).

##### Expression and Purification of Recombinant Human MUC5B N-terminal Domains

MUC5B (GenBank^TM^ accession number NM_002458) N-terminal constructs were created consisting of D1-D2-D′-D3 (NT5B) (residues 26–1304) (8), D1 (residues 26–424), D1-D2 (residues 26–793), D2-D′-D3 (residues 425–1304), and D3 (residues 894–1304) (see Fig. 3*a*). Recombinant proteins were expressed with an N-terminal His_6_ tag using the mammalian episomal expression vector pCEP-His in 293-EBNA (23) and A549 cells (human lung carcinoma cells). Conditioned medium was collected from stably transfected 293-EBNA cells and transiently transfected A549 cells and analyzed by SDS-PAGE and Western blots probed with anti-His antibody. Recombinant protein was purified by nickel-affinity chromatography using a 1-ml HisTrap FF column followed by size fractionation on Superose 6 (10/300 column eluted in 25 mm Tris, 10 mm NaCl, pH 7.4) and anion exchange chromatography on a Resource Q column (1 ml; eluted with a gradient of 0–0.5 m NaCl in 25 mm Tris, pH 7.4). Protein identity and purity were confirmed by tandem mass spectrometry (MS).

##### Equilibrium Dialysis to Investigate the Interaction between Intact Native MUC5B and Calcium

For equilibrium dialysis, samples of MUC5B (70 μl at 80 μg/ml) in Hepes-buffered NaCl (10 or 100 mm NaCl) were dialyzed in Dispo-Equilibrium Dialyzer^TM^ (molecular mass cut-off 10 kDa) against Hepes-buffered NaCl (70 μl) containing different concentrations of ^45^CaCl_2_. After 20 h at room temperature, radioactivity was determined in aliquots of the sample and diffusate by scintillation counting. Data were analyzed with GraphPad Prism 5.0 software, and curves were fitted to a single binding site or multiple binding sites.

##### Inhibition of Calcium-dependent Assembly of Intact MUC5B

Diffusion measurements on MUC5B were obtained from confocal fluorescence recovery after photobleaching experiments performed as described previously (19, 22). The concentration dependence of MUC5B self-diffusion was determined with FITC-mucin solutions (0.05–0.2 mg/ml) in 0.1 m NaCl, 20 mm Tris, pH 7.4, with and without 20 mm CaCl_2_. The inhibition of MUC5B self-diffusion by polypeptide specific antisera (dilution of 1/50) was determined with FITC-mucin (0.1 mg/ml) in 0.1 m NaCl, 20 mm Tris, pH 7.4, with and without 10 mm CaCl_2_. Rabbit polyclonal antisera were raised against peptide sequences in nontandem repeat regions of MUC5B (positions of the immunizing peptides on MUC5B are shown in Fig. 2*d*). MAN-5BI and MAN-5BIII have been described previously (24, 25). MAN5B-VI (D1), MAN5B-VII (D2), and MAN5B-VIII (D3) were tested for reactivity against expressed N-terminal D-domains by Western blotting (see Fig. 2*e*).

##### Size Exclusion Chromatography Multiangle Laser Light Scattering Analysis (SEC-MALLS)

For SEC-MALLS analysis, proteins were incubated overnight at 4 °C, in 5 mm CaCl_2_, 5 mm MgCl_2_ or 5 mm EGTA at pH 7.4 or pH 6 and then applied to a Superose 6 10/300 column in 25 mm Hepes, 150 mm NaCl (with CaCl_2_, MgCl_2_, or EGTA at pH 7.4 or pH 6 at room temperature; *n* = 3). Column eluents passed through an inline DAWN EOS laser photometer and an Optilab rEX refractometer with quasi-elastic light scattering dynamic light scattering attachment. Analysis was performed using ASTRA version 6 software.

##### Electron Microscopy and Image Analysis

For transmission electron microscopy (TEM) and image analysis, protein samples (∼10–20 μg/ml) were negatively stained in 2% (w/v) uranyl acetate. TEM data were recorded on a Tecnai BioTwin at 100 kV under low dose conditions. Images were recorded on a Gatan Orius CCD camera at 3.5 Å/pixel. All image processing was performed using EMAN2 (26) on data that were low pass Gaussian-filtered to 20 Å resolution, using strategies described previously (27). Particles were selected into 72-pixel (NT5B monomer) or 144-pixel (NT5B dimer and D2-D′-D3 complexes) boxes using selective swarm parameters in E2Boxer. All datasets contained ∼5000 unique particles. Following class averaging, preliminary models were generated to assess symmetry. The dimer-enriched sample had a clear C2 symmetry, and this was applied to all subsequent processing. Following five rounds of iterative refinement, the resolution was determined using FSC-0.5 criteria (26). Hydrodynamic parameters were determined with the HYDROMIC software (28).

##### Small Angle X-ray Scattering (SAXS)

SAXS data were collected on NT5B protein in 25 mm Tris, 200 mm NaCl, pH 7.4, at the P12 beam line (Petra-III (Deutsches Elektronen Synchrotron (DESY), Hamburg, Germany)). Data were collected at 10 °C using a European Molecular Biology Laboratory/European Synchrotron Radiation Facility (EMBL/ESRF) new generation automated sample changer. The scattering intensities were recorded using a Pilatus 2M pixel x-ray detector (DECTRIS) with sample-to-detector distance of 3.1 m (*q*-range 0.008–0.47 Å^−1^). The two-dimensional data were integrated and reduced as described previously (29). Data were analyzed using the ATSAS 2.5.1 software (EMBL Hamburg). An average model was produced from 10 *ab initio* structures using the DAMAVER software (30). Hydrodynamic parameters for the models were determined using HYDROPRO version 7.C (31).

##### Analytical Ultracentrifugation

The sedimentation coefficients of NT5B incubated in 5 mm CaCl_2_ or 5 mm EGTA at pH 7.4, pH 6, or pH 5 were determined from velocity experiments using the Optima XL-A ultracentrifuge (Beckman Instruments). Samples (*n* = 3) were centrifuged in a double sector cell at 35,000 rpm, taking 200 scans at 1.5-min intervals at 280 nm, at 20 °C. Sedimentation coefficients were determined using SedFit version 13.0b (32).

## RESULTS

### 

#### 

##### Calcium Binding to Native MUC5B

**FIGURE 1.**
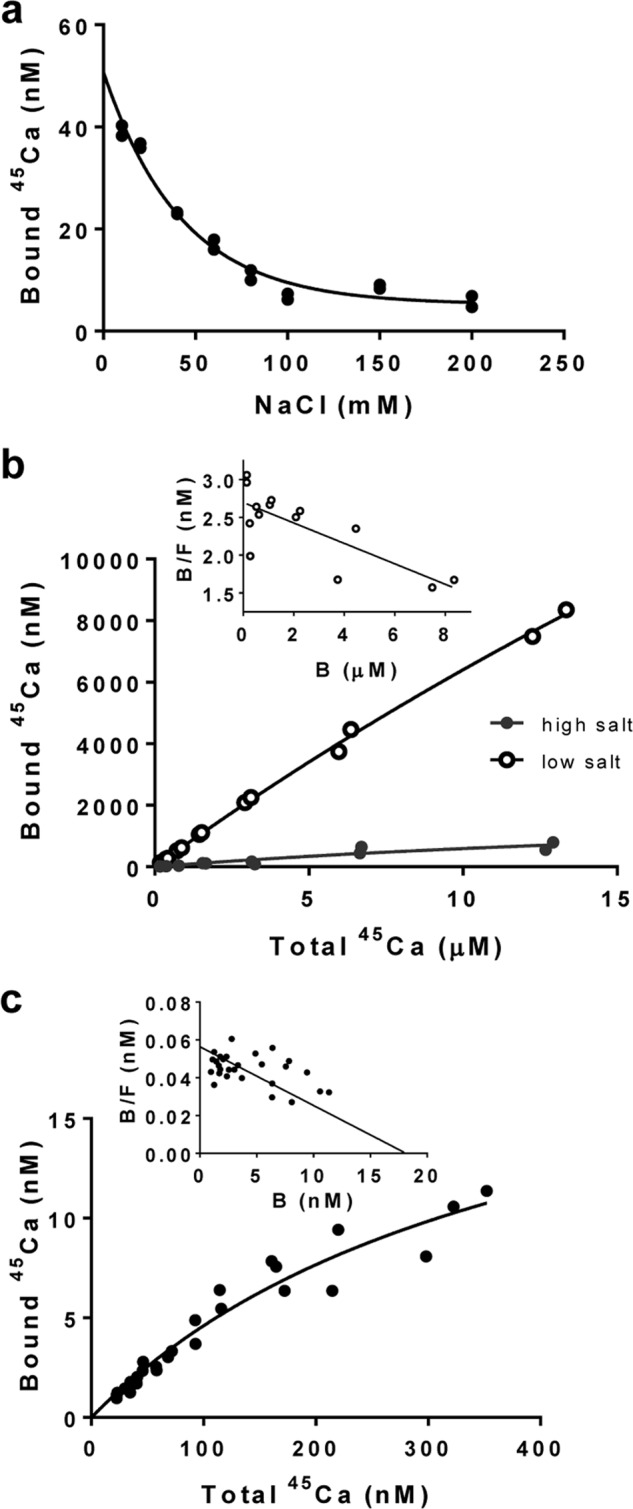
**Calcium binding to native MUC5B.** *a*, NaCl titration of the bound ^45^Ca to MUC5B by equilibrium dialysis. *b*, comparison of ^45^Ca binding to MUC5B in 10 mm (low salt; *open circles*) and 100 mm (high salt; *filled circles*) NaCl buffers. *Inset*: Scatchard plot of the ^45^Ca binding of MUC5B in 10 mm NaCl. *c*, More extensive analysis of ^45^Ca binding to MUC5B in 100 mm NaCl buffer. *Inset*: Scatchard plot of the ^45^Ca binding of MUC5B in 100 mm NaCl. B/F is the ratio of bound to free ^45^Ca.

We characterized ^45^Ca binding to native MUC5B by equilibrium dialysis, and to distinguish between specific (19) and nonspecific interaction (17, 18, 33, 34), binding was determined with increasing NaCl concentration (Fig. 1*a*). Most of the calcium binding to MUC5B was found to be salt-dependent and was lost in the presence of 100 mm NaCl, but there remained a small salt-independent fraction (Fig. 1, *a* and *b*). Characterizing the ^45^Ca binding in low salt (10 mm NaCl) showed that the amount bound was proportional to the calcium concentration and did not saturate at the conditions tested (Fig. 1*b*). This main fraction of calcium binding, which is likely attributed to ionic interactions with charged carboxyl and sulfate groups on mucin glycans, was associated with many low affinity binding sites (∼1680 sites/mol; *K_D_* ∼ 74 μm; Fig. 1*b*, *inset*). In contrast, the small fraction of salt-independent calcium binding (in 100 mm NaCl) was quickly saturated (Fig. 1*c*), and Scatchard analysis showed higher affinity binding to a single site per mucin monomer (Fig. 1*c*, *inset*; ∼0.72 site/mol in the sample tested; *K_D_* ∼0.4 μm).

##### Calcium-dependent Organization of Native MUC5B

**FIGURE 2.**
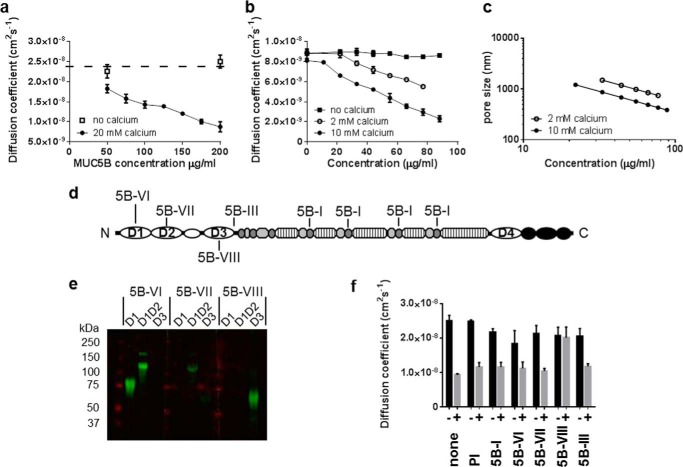
**The effect of calcium on native MUC5B self-diffusion.** *a*, the concentration dependence of FITC-MUC5B lateral self-diffusion in the absence (*open circles*) or presence (*filled circles*) of 20 mm CaCl_2_. *b*, the lateral diffusion of microspheres (499 nm) in a range of concentrations of native MUC5B containing 0 mm (*filled squares*), 2 mm (*open circles*), or 10 mm (*filled circles*) CaCl_2_. The *solid lines* for 2 and 10 mm CaCl_2_ show the fit to the following equation (*D* = *D*_0_ exp(−β *c*^υ^) using least squares analysis. *c*, the calculated average pore size ξ determined from the tracer diffusion measurements with 499-nm microspheres over a range of concentrations of native MUC5B solution containing 2 mm (*open circles*) and 10 mm (*filled circles*) CaCl_2_. The ξ values were calculated from the equation ξ = (*d*/β*) c*^−υ^ using the values of β and υ derived from the equation above. *d*, graphic of MUC5B (D-domains (*white ovals*), glycosylated domains (*hatched boxes*), Cys-domains (*gray ovals*), and other C-terminal domains (*black circles*) (8) highlighting the positions of the peptides used as immunogens for MAN5B-I, 5B-III, 5B-VI, 5B-VII, and 5B-VIII. *e*, Western blot analysis of N-terminal subdomain proteins (D1, D1-D2, and D3) showing the specificity of antisera that were raised against peptides from specific regions of MUC5B (MAN5B-VI, 5B-VII, 5B-VIII). *f*, the lateral self-diffusion of FITC-MUC5B (0.1 mg/ml) in 0 mm (*black bars*) or 10 mm CaCl_2_ (*gray bars*)with different MUC5B antisera or preimmune serum (*PI*). *Error bars* show the S.D. to the calculated mean for five replicates. In *panels a*, *b*, and *f*, measurements were performed in 0.1 m NaCl, 20 mm Tris, pH 8.0, at 25 °C, and the *error bars* show the S.D. to the calculated mean for five replicates.

To investigate the effect of this high affinity calcium binding on MUC5B macromolecular organization, we performed confocal fluorescence recovery after photobleaching experiments. Calcium caused a major decrease in the self-diffusion of purified FITC-MUC5B (50–200 μg/ml; Fig. 2*a*). The supporting electrolyte (0.1 m NaCl) in these experiments was sufficient to rule out weak ionic interactions and link the effects to calcium binding at the higher affinity site. These major changes in physical properties with an apparent increase in MUC5B molecular mass, a decrease in self-diffusion, and lowered gel porosity (Fig. 2, *b* and *c*) are compatible with the generation of reversible cross-links between polymeric MUC5B chains.

To investigate which protein domain of the MUC5B mucin was responsible for the calcium-dependent interactions, antisera raised against peptides from specific regions of MUC5B were tested as inhibitors of the calcium effects on MUC5B self-diffusion (Fig. 2, *d–f*). Only one antiserum (MAN5B-VIII), which was raised against a unique peptide located in the D3-domain (Fig. 2*f*), completely inhibited the calcium effect on MUC5B self-diffusion, whereas four other antisera to peptides in D1-, D2-, and Cys-domains and between D3 and the glycosylated domain had no effect (Fig. 2*f*). As MAN5B-VIII completely blocked the noncovalent calcium-dependent interaction between the MUC5B mucins, its peptide antigen in the D3-domain must be close to the calcium-dependent site mediating MUC5B self-association.

##### Expression and Purification of N-terminal MUC5B Protein Domains

**FIGURE 3.**
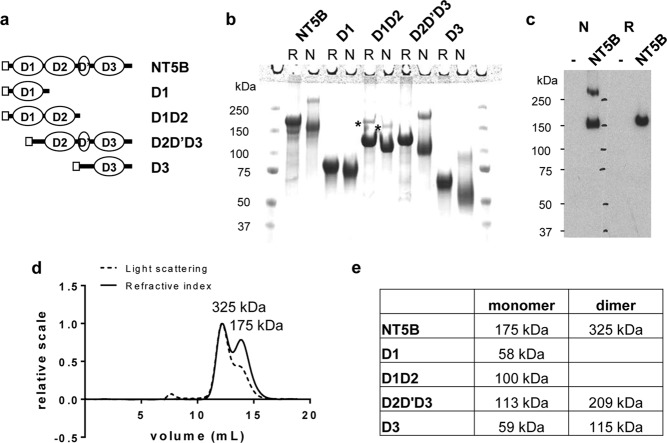
**Expression of recombinant MUC5B N-terminal protein domains.** *a*, schematic showing the domains of N-terminal constructs of MUC5B. *b*, the expressed N-terminal construct (NT5B; D1-D2-D′-D3-domains) and subdomains (D1, D1-D2, D2-D′-D3, and D3) were analyzed by SDS-PAGE (reduced (*R*) and nonreduced (*N*)). Different NT5B protein preparations yielded varying proportions of monomer and dimer. The highlighted reduction-insensitive band detected in the D1-D2 preparation (*) was identified as desmoplakin by tandem MS. *c*, Western blot analysis of conditioned medium, reduced (*R*) and nonreduced (*N*), from NT5B expressed transiently in A549 cells (NT5B) and untransfected cells (−), probed with anti-His antibody. A549 cells expressed NT5B as a monomer and disulfide-linked dimer, which was comparable with NT5B expressed from 293-EBNA cells. *d*, SEC-MALLS analysis of NT5B showing light scattering (*dashed line*) and refractive index (*solid line*). *e*, molecular mass values of NT5B and expressed N-terminal MUC5B subdomains determined from SEC-MALLS analysis.

The N-terminal region of MUC5B is the site for the covalent disulfide head-to-head linkages that are a recognized core feature of mucin polymer formation. Therefore, to gain further insight into the role of the N-terminal domains in the intracellular assembly, packaging, and organization of MUC5B, we stably expressed the recombinant N-terminal domain of MUC5B (NT5B), which included D1-D2-D′-D3-domains and related truncated constructs in 293-EBNA cells (Fig. 3*a*). The expressed MUC5B proteins were purified from conditioned cell medium and analyzed by SDS-PAGE (Fig. 3*b*), and tandem mass spectrometry confirmed their identity. All of the constructs were expressed and secreted by the 293-EBNA cells. The tendency of some of the expressed constructs to form disulfide-bonded dimers was an initial distinguishing feature (Fig. 3*b*). Only the constructs containing the D3-domain formed dimers in addition to monomers in the secreted products. The whole N-terminal (NT5B) protein was expressed as a monomer (∼150 kDa) and a dimer (∼300 kDa). The D2-D′-D3 protein and D3 protein were also expressed as monomers (∼110 and ∼50 kDa, respectively) and dimers (∼220 and ∼100 kDa, respectively), whereas the D1 and D1-D2 proteins were expressed only as monomers (∼75 and ∼110 kDa, respectively). These results confirmed that the D3-domain-containing constructs expressed in 293-EBNA cells replicated the intermolecular disulfide links observed in intact MUC5B.

**FIGURE 4.**
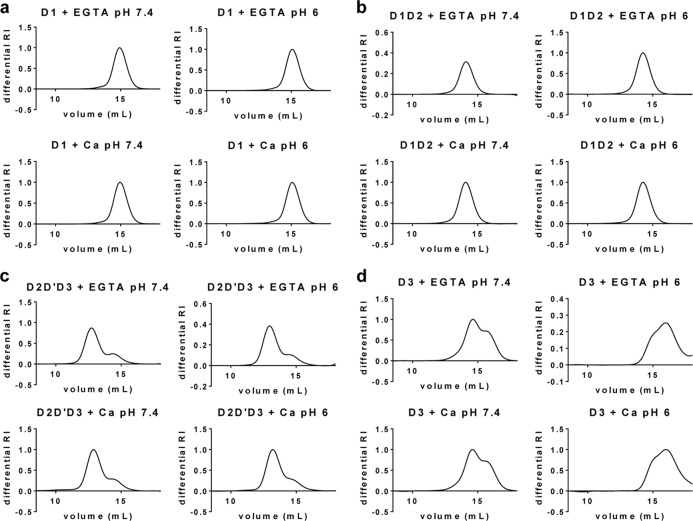
**SEC-MALLS analysis of N-terminal subdomains of MUC5B.** N-terminal subdomains of MUC5B were incubated with 5 mm CaCl_2_ or 5 mm EGTA, pH 7.4 or pH 6, and analyzed by SEC-MALLS. Representative graphs show the differential refractive index (*RI*). *a–d*, chromatographs for D1 (*a*) and D1-D2 (*b*) constructs showed one peak corresponding to the monomer, whereas D2-D′-D3 (*c*) and D3 (*d*) constructs showed two peaks corresponding to the dimer (*first peak*) and monomer (*second peak*). There was no effect on the chromatographic profiles of the subdomains D1, D1-D2 and D2-D′-D3 in the presence of calcium at low pH. However, D3 protein showed an effect from low pH in the presence and absence of calcium (*d*), indicating a potential pH-dependent aggregation.

Biophysical characterization of NT5B (Fig. 3*d*) and the other N-terminal protein subdomains (D1, D1-D2, D2-D′-D3, and D3; Fig. 4, *a–d*, respectively) was performed by SEC-MALLS to provide size fractionation and absolute determination of molecular mass. SEC-MALLS results confirmed that MUC5B proteins containing the D3-domain were expressed only as monomer and dimer, and those without D3-domain were expressed only as monomers (Fig. 3, *d* and *e*). The relative proportion of monomer and dimer varied between NT5B preparations. However, for each preparation studied, there was no suggestion of an equilibrium as the relative proportions of monomer and dimer did not vary with concentration, which was compatible with dimers being disulfide-bonded. This pattern of expression was not unique to 293-EBNA cells as the NT5B construct transiently transfected into A549 cells (a mucin-producing cell line) was also expressed and secreted as a monomer and disulfide-linked dimer (Fig. 3*c*).

##### Structural Analysis of N-terminal MUC5B Monomer and Dimer

**FIGURE 5.**
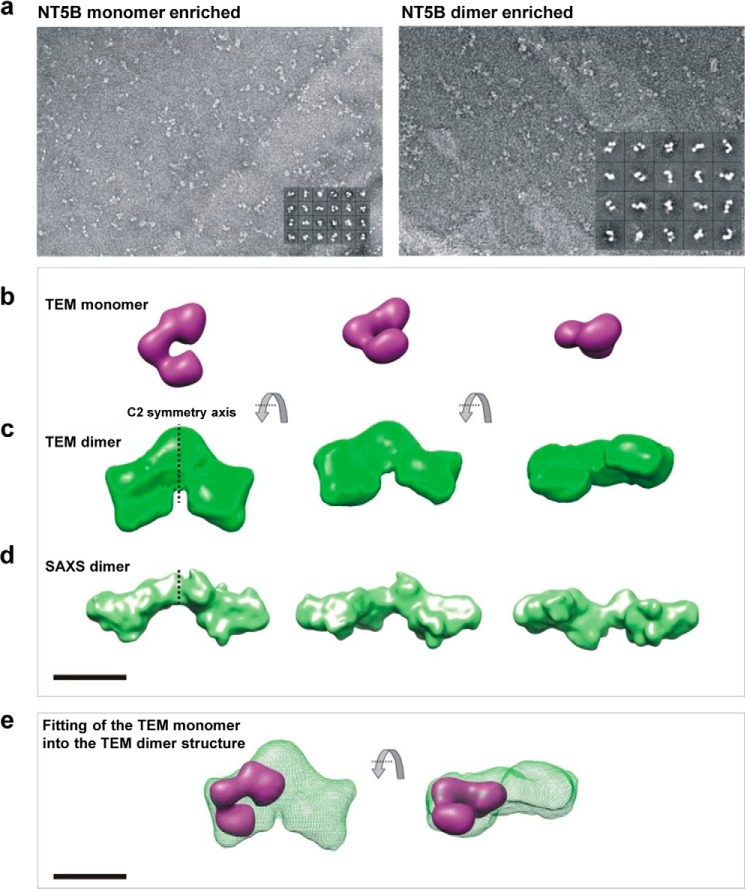
**Structural analysis of monomer and dimer-enriched NT5B.** TEM analysis of monomer and dimer-enriched NT5B is shown. *a*, negatively stained particle averages for NT5B monomer (*left panel*) and NT5B dimer (*right panel*). *Insets* show representative class averages. *b*, the three-dimensional model of NT5B monomer, in three orientations. *c*, the three-dimensional model of NT5B dimer, in three orientations. *d*, SAXS analysis of the solution structure of NT5B dimer shown in three orientations. *e*, the monomer modeled into the dimer structure. *Scale bars* are 100 Å.

**FIGURE 6.**
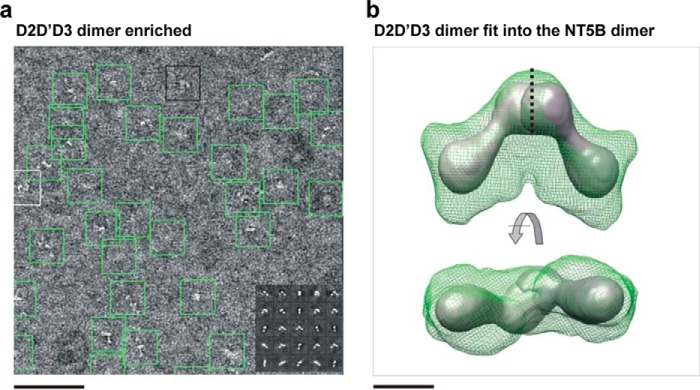
**TEM analysis of dimeric D2-D′-D3 MUC5B.** *a*, a sample field of negatively stained ”raw“ D2-D′-D3 particles. *Scale bar* = 100 nm. The *inset* shows examples of projection averages determined from the raw data. *b*, three-dimensional model of dimeric D2-D′-D3 in two orientations showed N-terminal dimers formed between homotypic D3-domains. *Scale bar* = 50 Å.

To investigate the structure of the purified NT5B monomer and dimer, we performed single particle TEM. Samples enriched in either monomeric or dimeric NT5B were separated by size exclusion chromatography and imaged in negative stain (Fig. 5*a*). For each sample, a three-dimensional reconstruction was calculated from ∼5000 particles. The structure of the monomer-enriched NT5B had three distinct lobes, potentially corresponding to the three D-domains, which formed an elongated but bent shape (Fig. 5*b*) with dimensions of 13 × 9.5 × 7 nm. The structure visualized from the dimer-enriched NT5B sample was approximately twice the size of the monomer with dimensions 24 × 17 × 9 nm. The dimer also had an extended, boomerang-like shape, but with a more pronounced central region (Fig. 5, *c* and *e*). To confirm that the D3-domain was located centrally within the dimer, a construct (D2-D′-D3) lacking the D3-domain was analyzed (Fig. 6), and this also had a boomerang shape. Together these data support the model that dimerization of NT5B is mediated by disulfide links between the D3-domains and these form the junction between monomers in the TEM dimer image. The HYDROMIC software (28) was utilized to calculate the hydrodynamic properties of the TEM monomer and dimer structures, and these predicted parameters showed good agreement with the values determined experimentally by sedimentation velocity analysis (AUC) (Table 1).

**TABLE 1:** **Hydrodynamic parameters of NT5B monomer and dimer** Hydrodynamic parameters were determined from NT5B in solution (SEC-MALLS, AUC, and SAXS) and EM structures. ND,- not determined.

NT5B	Mass	*S*	*R_h_*	*R_g_*
	*kDa*		*nm*	*nm*
Monomer	175 ± 17.8*^a^*	6.37 ± 0.03*^b^*	5.93 ± 0.31*^a^*	ND
Monomer (EM)	ND	5.7*^c^*	6.1*^c^*	5.16*^c^*
Dimer	325 ± 6.5*^a^*	8.99 ± 0.04*^b^*	10.05 ± 0.34*^a^*	ND
Dimer (SAXS)	318*^d^*	9.89*^d^*	8.51*^d^*	7.66*^d^*
Dimer (EM)	ND	7.6*^c^*	9.4*^c^*	8.39*^c^*

*^a^* Derived from SEC-MALLS.

*^b^* SedFit determined the best-fit for mass-independent frictional ratio.

*^c^* HYDROMIC calculated the theoretical standard hydrodynamic parameters for EM structures.

*^d^* HYDROPRO calculated the theoretical standard hydrodynamic parameters for SAXS structure.

**FIGURE 7.**
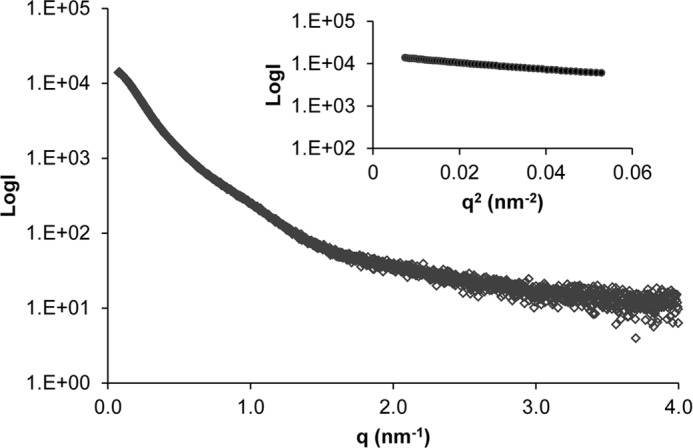
**SAXS data for NT5B dimer.** Shown are SAXS data collected for the NT5B dimer showing the scattering intensity plotted as a function of *q. Inset*: Guinier plot indicating *R_g_* = 7.7 nm.

SAXS data were collected for dimer-enriched samples of NT5B at ∼10 mg/ml concentration at the synchrotron radiation source, Petra III. The radius of gyration (*R_g_*) for the dimer obtained from the Guinier plot was 7.7 nm (Fig. 7), and the maximum particle dimension was estimated at 26.6 nm using GNOM (35) (data not shown). *Ab initio* bead models were generated using the DAMMIN program (36). Ten simulations of DAMMIN were computed to determine the common structural features and averaged to calculate the three-dimensional structure using the DAMAVER software (30) (Fig. 5*d*). The SAXS model generated, with dimensions 26 × 11 × 9 nm, closely resembled the structure observed in TEM (Fig. 5*c*).

The theoretical sedimentation properties of the SAXS structure were calculated using the HYDROPRO software (31), and the predicted values were also compared with those determined experimentally (Table 1). This confirmed the similarity between models derived from SAXS and TEM data and their compatibility with the AUC analysis (Table 1).

##### Effect of Calcium and pH on N-terminal MUC5B

**FIGURE 8.**
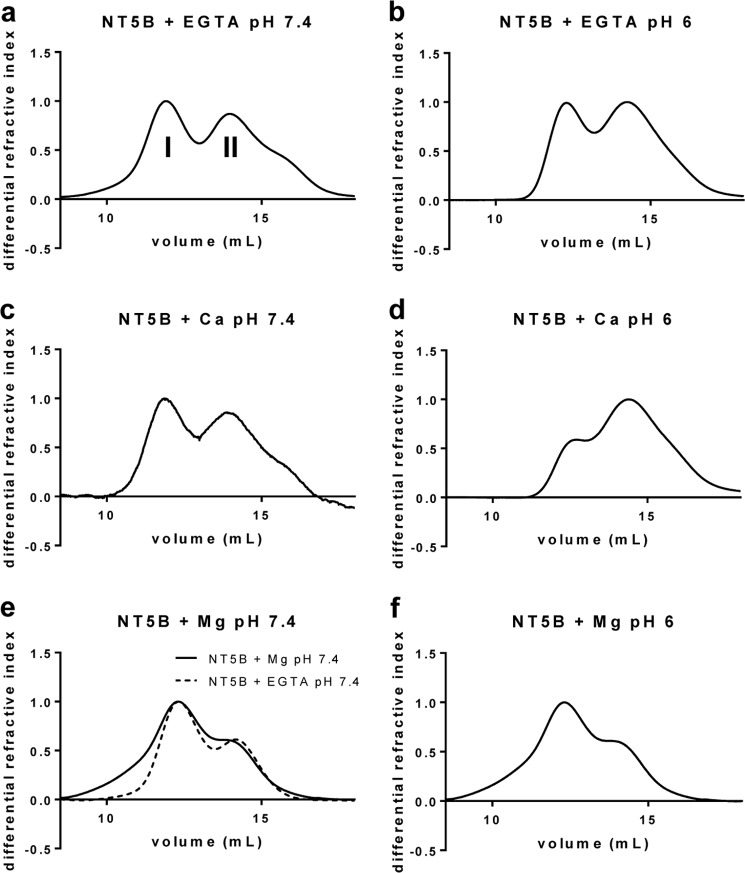
**pH-dependence of calcium-mediated aggregation of the intact N-terminal domain of MUC5B.** *a–f*, recombinant NT5B was incubated with 5 mm EGTA at pH 7.4 (*a*) and pH 6 (*b*), 5 mm CaCl_2_ at pH 7.4 (*c*) and pH 6 (*d*), and 5 mm MgCl_2_ at pH 7.4 (*e*) and pH 6 (*f*), and analyzed by SEC-MALLS. Representative graphs show the differential refractive index (*n* = 3). Chromatographs showed two peaks corresponding to the dimer (*peak I*) and monomer (*peak II*). The refractive index measurements showed an ∼15% reduction in the amount of NT5B protein recovered in the dimer peak in 5 mm CaCl_2_ at pH 6 as compared with 5 mm EGTA at pH 6. Also shown in *e* is the control for recombinant NT5B without MgCl_2_ (NT5B incubated with 5 mm EGTA at pH 7.4), which demonstrates that MgCl_2_ did not cause NT5B dimers to associate into higher order structures.

During biosynthesis MUC5B is formed into a polymer in the acidic compartments of the Golgi and subsequently packed inside secretory granules in the presence of a high Ca^2+^ concentration prior to secretion. How MUC5B polymers are organized within the granules has yet to be determined. To investigate the effect of Ca^2+^ and pH on the N-terminal domain of MUC5B, NT5B was analyzed by SEC-MALLS after incubation with and without calcium (CaCl_2_ or EGTA) at pH 7.4 and at pH 6.0 (Fig. 8, *a–d*). NT5B eluted as a dimer (*peak I*) and monomer (*peak II*) under all conditions. However, in the presence of Ca^2+^ at pH 6.0, there was a loss in recovery of NT5B from the column, which appeared preferentially to be a loss of dimer (Fig. 8*d*). The effect was specific for calcium as there was no comparable effect with MgCl_2_ (Fig. 8, *e* and *f*). These data suggested that calcium caused NT5B dimers to associate into higher order structures, which were retained on the column.

In contrast, shorter constructs including D3 and the individual N-terminal subdomains showed no comparable loss on the column. However, D3 protein showed an altered elution profile at low pH in both the presence and the absence of Ca^2+^ (Fig. 4*d*). These results suggested that calcium-dependent association of the dimer required D3 in the context of the full N terminus of MUC5B. The results also suggested that there are two effects on the D3-domain with low pH (*a*) causing a change in its conformation and (*b*) potentiating the calcium-mediated association of N-terminal dimers.

**FIGURE 9.**
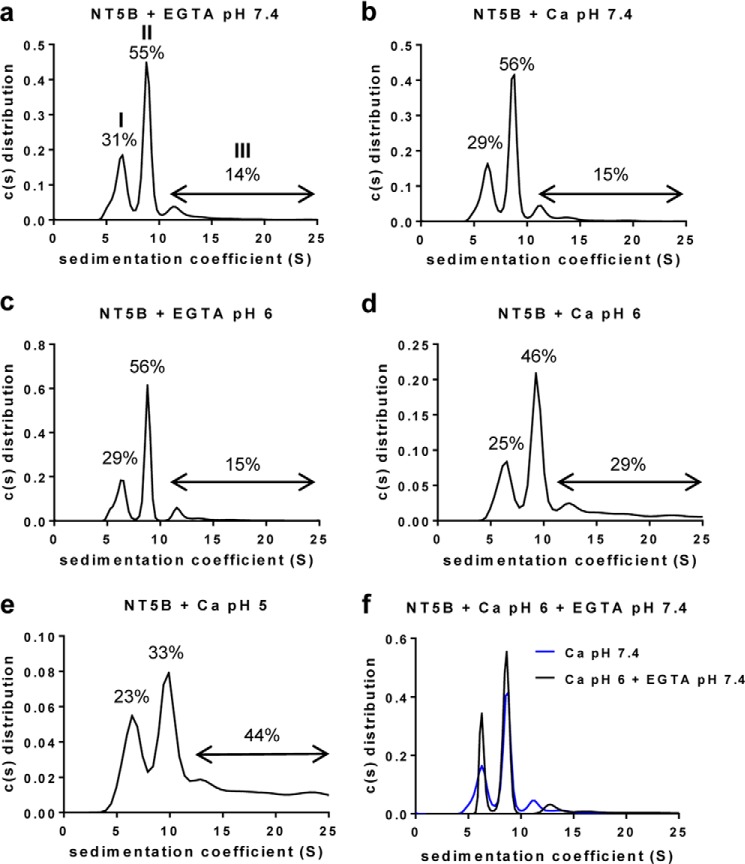
**pH-dependent, calcium-mediated multimerization of disulfide-linked N-terminal MUC5B dimers.** *a–d*, recombinant NT5B was incubated with 5 mm EGTA at pH 7.4 (*a*) and pH 6 (*c*) and 5 mm CaCl_2_ at pH 7.4 (*b*) and pH 6 (*d*), and analyzed by AUC to identify multimer formation (*n* = 3 for each combination). Representative profile showed three peaks corresponding to a monomer (*peak I*), a dimer (*peak II*), and a larger multimer (*peak III*). *e*, the calcium-mediated multimerization was studied at lower pH, and NT5B was incubated with 5 mm CaCl_2_ at pH 5. Percentage values of the different NT5B species were calculated from the area under the peaks. *f*, NT5B multimers formed at pH 6 in 5 mm CaCl_2_ were incubated with 10 mm EGTA at pH 7.4 overnight at 4 °C and analyzed by AUC (*n* = 3). The NT5B multimers were partially dissociated following incubation with EGTA at pH 7.4.

To investigate the apparent loss of the calcium-dependent NT5B multimers, we performed sedimentation velocity AUC (Fig. 9). Sedimentation profiles were obtained for NT5B incubated with 5 mm CaCl_2_ or 5 mm EGTA at pH 7.4 and at pH 6.0, and the sedimentation coefficient for each species present was determined (Fig. 9, *a–d*). Sedimentation profiles analyzed using SedFit (32) for NT5B with Ca^2+^ at pH 7.4 or for NT5B with EGTA at pH 7.4 and at pH 6 were identical, showing two major peaks corresponding to the monomer (*peak I*) and dimer (*peak II*) and a minor peak (*peak III*) showing a very low proportion of a larger multimeric species (Fig. 9, *a* and *b*), and the EGTA control at pH 6.0 was also similar (Fig. 9*c*). In contrast, the sedimentation profile for NT5B with Ca^2+^ at pH 6 showed an increased sedimentation rate for the dimer (*peak II*) and an increase in the multimeric fraction (*peak III* ∼29% of total; Fig. 9*d*). The increased sedimentation rate suggested a change in conformation of NT5B dimer at pH 6 to a more compact structure, and this was accompanied by the generation of larger multimers forming additional peaks with higher sedimentation coefficients (Fig. 9*d*). Further experiments at pH 5.0 in the presence of Ca^2+^ showed an even higher proportion (44% of total) of higher order multimeric species (Fig. 9*e*). These results confirmed that in the presence of Ca^2+^, multimers of N-terminal MUC5B were formed at pH 5.0 and pH 6.0. It was noticeable that they appeared to form only between dimers because the proportion of monomer (*peak I*) remains similar at the different pH values, but the proportion of dimer (*peak II*) decreased (Fig. 9, *d* and *e*). The effect of calcium at low pH on the sedimentation of the dimer and the formation of larger multimers was shown to be reversible as the addition of 10 mm EGTA and adjustment of the pH to 7.4 resulted in the return of the sedimentation profile to that observed in the absence of Ca^2+^ at pH 7.4 (Fig. 9*f*). These results established that MUC5B N-terminal domains provided not only the site for intermolecular disulfide bonds involved in the assembly of linear MUC5B polymeric chains, but also a site for reversible pH-sensitive and calcium-dependent intermolecular association between these polymeric chains.

## DISCUSSION

**FIGURE 10.**
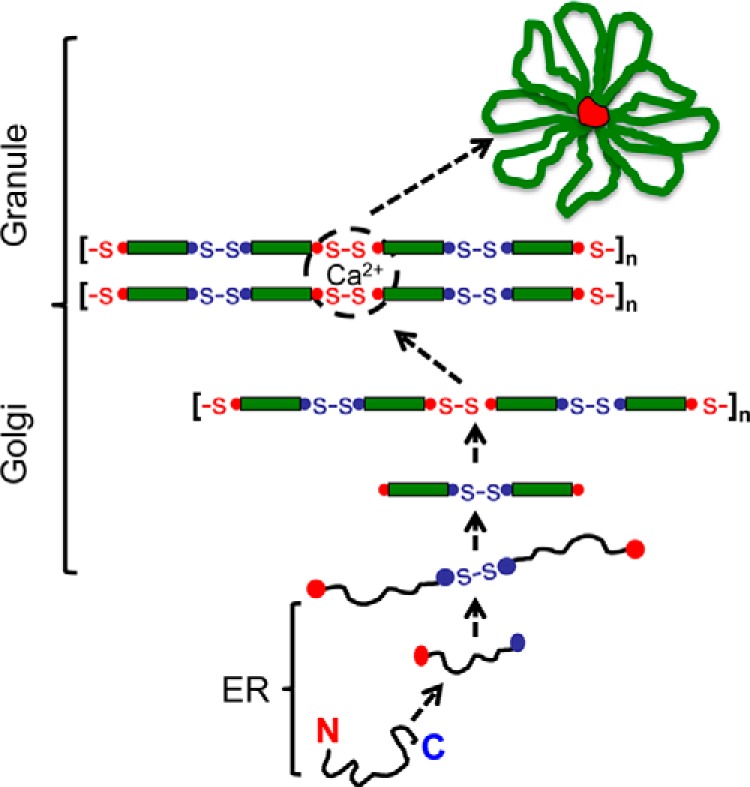
**Model for MUC5B intracellular assembly and packaging.** The MUC5B polypeptide undergoes dimerization via C-terminal to C-terminal domain (*blue circles*) disulfide linkage in the endoplasmic reticulum (*ER*). After transport to the Golgi, the dimer is glycosylated prior to assembly into linear polymers via N-terminal to N-terminal domain (*red circles*) disulfide links. As pH decreases and free calcium concentration increases across the secretory pathway, a conformational change occurs in the dimeric N termini, and noncovalent, calcium-mediated interactions between dimeric N termini become active. These focal links may occur within and between chains and organize MUC5B polymers for intragranular packaging. These focal links might represent the ”proteinaceous nodes“ observed in TEM images of freshly secreted MUC5B (21). Calcium binding to the charged glycans in the mucin domains (*green rectangles*) and its effect on mucin organization are not depicted.

This investigation brings new insight into the molecular events that characterize key stages in the process of intracellular assembly of MUC5B. Applying a range of biophysical methods has enabled unambiguous identification of disulfide-linked molecular intermediates and characterization of their noncovalent, calcium-dependent interactions, allowing a new model of polymeric mucin assembly and packaging to be derived (Fig. 10). The results shown here with MUC5B are compatible with a basic model of assembly comparable with vWF, in which mucin monomers are disulfide-linked C-terminal to C-terminal domain in the rough endoplasmic reticulum, before translocation to the Golgi, where they are disulfide-linked N-terminal to N-terminal domain, to form linear arrays. However, we have now characterized an additional reversible association between MUC5B N-terminal D3-domains that changes the organization of the linear polymeric chains through calcium-mediated cross-links. These are proposed to form an essential part of the mechanism involved in MUC5B condensation and packing that characterizes its organized storage in secretory granules, and the uncoupling of these cross-links would favor the rapid and efficient unpacking and expansion of MUC5B as degranulation releases the mucus gel to fulfill its physiological function.

Biophysical and TEM analyses presented here showed that recombinant N-terminal domains of MUC5B form dimers, through disulfide linkage between D3-domains. This mode of assembly of the intact mucin would create a linear polymer. Studies on MUC5AC have also concluded that the assembled C-terminal linked dimers form disulfide links through their N-terminal domains to form dimers of dimers (16), also resulting in linear polymers (37). In contrast, experiments, principally on the intestinal mucin MUC2 (but also with porcine submaxillary mucin), have been interpreted to propose that for these mucins, C-terminal linked dimers are assembled to form disulfide-linked N-terminal trimers, which would generate polymers with a highly branched, network-like structure (14, 38). There was no evidence in our results with MUC5B for the formation of trimeric species. It remains to be resolved whether all the gel-forming mucins share a common assembly mechanism or whether, despite their close structural homology, they have evolved these two quite distinct pathways of assembly.

The calcium-mediated interaction reported here implies that each cross-link between two MUC5B chains involves four N-terminal domains (two disulfide-linked pairs in each chain; Fig. 10). The molecular modeling identified in dimers of the expressed N-terminal domains that the disulfide bridge caused close proximity of adjacent D3-domains, and as the D3-domain was identified as the site of the calcium-mediated cross-link, this generates a plausible model to provide a protein interaction interface involving four D3-domains each with a calcium-binding site. The precise topography of this interaction will require more analysis to generate a detailed structural model. There are further important features of the model of assembly predicted from this structural analysis. Firstly, the affinity of the MUC5B protein site for calcium (*K_D_* ∼ 0.4 μm) suggests that calcium occupancy of the binding site will be sensitive to physiological ranges of calcium concentration found in intracellular compartments. Thus in environments low in free calcium, such as the rough endoplasmic reticulum, there may be only limited calcium binding to MUC5B, whereas in late Golgi and secretory vesicles with high free calcium, the binding may approach saturation. The protein-protein interaction mediated by calcium will also be concentration-dependent, and thus cross-links between mucin chains will be maximized in the condensed state of MUC5B in secretory granules. The results also provide two other important pieces of evidence suggesting that conditions favoring calcium-mediated cross-links only occur late in intracellular assembly. Firstly, the formation of cross-links is enhanced at low pH (pH 5–6), which occurs physiologically in late Golgi and in secretory granules. The change in sedimentation of the NT5B dimer at pH 6.0 also suggested that a change in protein conformation at these pH values may be necessary to form cross-links. Secondly, the results with the expressed N-terminal domain of MUC5B showed that the dimer of the N-terminal domain formed calcium-mediated interactions, but not the monomer. This implies that MUC5B, prior to becoming N-terminal disulfide-linked, lacks the potential, even in the presence of calcium, for protein-protein interaction to form cross-links. These two factors would be important in providing a mechanism that would prevent the formation of cross-links in MUC5B until the N-terminal disulfide bridges are formed between D3-domains in the late Golgi, to complete the assembly of linear MUC5B chains. The high calcium concentrations and low pH found in secretory granules would also promote maximal cross-link formation as part of mucin condensation. The appearance of higher order multimers in addition to dimers of the NT5B construct at low pH with calcium may also suggest that under favorable conditions, such as in the secretory granule, calcium may facilitate the stacking of multiple chains through N-terminal D3-domain interactions, to provide additional order to the packaged mucin (Fig. 10). The extent to which this model of protein interaction helps drive mucin packaging in secretory granules will depend on many local factors including calcium concentration, pH, and also the effects of the large glycosylated regions, which were not assessed in this study. These new results may also provide a mechanism to interpret previous proposals that MUC5B in secretory granules was organized around “nodes” formed by interaction between N-terminal regions (21) and may also explain the network appearance in TEM images of native mucin preparations (39) and pH-dependent protein-mediated linkages that were postulated to be important for intragranular condensation of mucin (40).

A further important consequence of this model is that during release from the secretory granule, the sharp rise in pH and a decrease in Ca^2+^ concentration (20) would uncouple the multiple cross-links between MUC5B chains and provide an unfolding mechanism to generate the expanded mucin gel. However, in pathological situations, in particular in cystic fibrosis, where the extracellular pH is decreased and the calcium concentration is elevated, the failure to uncouple calcium cross-links could be a major factor affecting mucin expansion and compromising mucus properties (33, 34, 41–45).

## Footnotes

2 The abbreviations used are:

vWF: von Willebrand factor
EBNA: Epstein–Barr virus nuclear antigen
SEC-MALLS: size exclusion chromatography multiangle laser light scattering
TEM: transmission electron microscopy
SAXS: small angle x-ray scattering
AUC: analytical ultracentrifugation.
